# Eradication of hepatitis C virus infection in thalassemia patients in Iran using various treatment strategies

**DOI:** 10.1016/j.jve.2020.100006

**Published:** 2020-08-05

**Authors:** Meisam Moghimbeygi, Seyed Moayed Alavian

**Affiliations:** a Department of Mathematics, Faculty of Mathematics and Computer Science, Kharazmi University, Tehran, Iran; b Baqiyatallah Research Center for Gastroenterology and Liver Diseases, Baqiyatallah University of Medical Sciences, Tehran, Iran

**Keywords:** Hepatitis C, Thalassemia, Meta-analysis, Systematic review, Prevalence

## Abstract

**Background:**

Chronic hepatitis C virus (HCV) infection is considered as one of the leading causes of liver disease in thalassemic patients in Iran. Over 40% of the mortality in these patients is related to HCV.

**Objectives:**

The present study aimed at estimating HCV prevalence in thalassemic patients in Iran and to determine the number of infections until eradication is achieved.

**Methods:**

A meta-analysis approach was used to estimate the number of HCV-infected thalassemic patients in the country. The prevalence rate was measured using a modeling approach to predict the number of cases until eradication using several scenarios in terms of testing and treatment, in particular the use of direct acting antiviral drugs (DAAs).

**Results:**

With the advent of DAAs with a high rate of treatment success, HCV could be eradicated earlier than originally thought among this group of patients. Based on previous predictions the number of HCV-infected thalassemic patients would have been below 66 by 2020. However, according to our predictions, the number will be less than 10 when using DAAs.

**Conclusion:**

We believe that HCV eradication can be achieved in thalassemic patients with an increased life expectancy by funding DAA-based new treatment strategies. This has been exemplified in Alborz, Lorestan, and South Khorasan provinces with HCV eradication in this group of patients.

## 1 Background

Hepatitis C virus (HCV) infection is a global public health problem associated with liver-related morbidity and mortality due to disease progression.^1^ It is estimated that over 170 to 200 million people are HCV-infected with a prevalence ranging from 0.2% to 40% among countries.^2^^,^^3^ Although the risk for HCV acquisition with blood products remains present, donor screening and testing procedures have significantly reduced transmission. Chronic hepatitis follows acute HCV in 55–85% of patients with progression to cirrhosis in nearly 20% of them and development of hepatocellular carcinoma at a yearly rate of 3–5%.^4^

Patients with thalassemia major are more likely to suffer from blood transfusion-transmitted hepatitis. In fact, HCV is the most common cause of transfusion-transmitted infection among these patients with over 40% of HCV-related mortality.^5^ The incidence of hepatitis B virus (HBV) infection has decreased in this group of patients due to the increasing use of HBV immunization and its significant contribution to disease prevention.

Hepatitis C is still considered a serious problem in thalassemic patients due to the absence of an HCV prophylactic vaccine.^6^^,^^7^ Among those who had been transfused before the 1990's, HCV prevalence was reported to be proportional to the number of blood units received and approached 80% among adult patients.^8^^,^^9^ However, The risk of HCV infection post-transfusion infection decreased following the establishment of donor screening programs. However, mortality due to liver-related disease and cancer increased from 1990 due to the high hepatitis prevalence, although the HCV rate was reduced during 1980–1990 because of the donor screening and needle exchange programs.^10^ The screening program for blood donors was implemented in 1996 in the country. According to previous reports, patients who received blood transfusions before 1996 were 12.5 times more likely to be positive for HCV antibodies (Abs).^11^

Treatment with interferon (IFN) as standard therapy for chronic HCV infection has been used widely in Iran. Although relatively successful in the country, it has many shortcomings, such as poor tolerance, prolonged treatment course and suboptimal efficacy.

With this background in mind, we have aimed in this study to predict the number of HCV-infected thalassemic patients in the coming years by using a systematic data review and meta-analysis methodology as well as mathematical modelling. In addition, we have evaluated the effect of different parameters on the HCV eradication period. Our results can pave the way for rapid HCV elimination in thalassemic patients in the country when using the latest treatment developments.

## 2 Materials and methods

This study was conducted in two separate parts. First, we undertook a systematic review and meta-analysis to estimate HCV prevalence in thalassemic patients. Next, we inputted, the number of HCV-infected thalassemic patients into a model.

### 2.1 Data extraction and analysis

The study population consisted of thalassemic patients in Iran. The primary outcome was the presence of HCV Abs in blood samples, using an enzyme-linked immunosorbent assay (ELISA). A systematic review and meta-analysis were performed in SID, IranMedex, IranDoc, MagIran, MedLib, PubMed, ISI, Scopus, and Google Scholar to find relevant studies on HCV prevalence in the country during 1996–2015. Another study was performed during 2003–2006 in order to estimate HCV prevalence in 2003 in our mathematical model. The search was conducted using English keywords including “thalassemia” and “hepatitis C virus” or “HCV” or “chronic hepatitis C”, and their Persian equivalents with all possible combinations. In addition, titles and reference lists of the selected articles were reviewed as an additional search tool. Two researchers studied the extracted data independently and attempted to resolve differences if a conflict or inconsistency arose.

### 2.2 Predictive model

A mathematical model was designed for predicting the number of HCV-infected thalassemic patients, based on estimated parameters, as well as those reported in the literature, using Microsoft Excel (Microsoft Corporation, Redmond, WA, USA).

#### 2.2.1 Thalassemia mortality rate

Generally speaking, mortality rate in thalassemic patients in the country has been a function of disease, while use of drugs, accidents, and other natural causes of mortality play no significant role. According to a previous report,^14^ the annual mortality rate was estimated at 1.02 per 100 people.

#### 2.2.2 Total thalassemic population

We attempted to determine the total number of thalassemic patients in order to predict the number of HCV-infected cases. The total number of patients registered by the Iranian Ministry of Health and Medical Education reached nearly 18,500 people in 2007.^12^ A forward and backward selection model was used to calculate the number of thalassemic patients in other years. In this model, estimates were calculated by adding the number of births and reducing it by the number of deaths.

#### 2.2.3 HCV prevalence in thalassemic patients

The HCV prevalence in thalassemic patients was determined, using a meta-analysis and systematic review methodology. In our model, the prevalence rate during 2003–2006, which was based on a random effect model, was determined to estimate the HCV prevalence in 2003. In addition, a recursive model was constructed to predict the number of HCV-infected thalassemic patients annually. This was equivalent to the total number of new cases minus the number of treated cases. Mortality was measured as follows:

Number of HCV infections in year (x) ​= ​Number of HCV infected people until year (x) - treated cases - mortality rate.where the number of HCV infections in year (x) is the number of HCV infections in year (x-1) ​+ ​(number of thalassemic patients until year (x) - number of HCV infections in year (x-1)) ​× ​incidence rate; number of treated cases is the number of HCV infections in year (x-1) ​× ​treatment rate ​× ​diagnosis rate; and mortality rate is the number of HCV infections in year (x-1) ​× ​mortality rate due to HCV in thalassemic patients ​× ​thalassemia mortality rate.

#### 2.2.4 HCV incidence

Since the data related to the HCV incidence was unavailable, it was estimated on the basis of our model. Accordingly, the incidence rate was determined using a recursive mathematical model by measuring the number of HCV infections in thalassemic patients registered at the Iranian Ministry of Health and Medical Education at three times-points.

#### 2.2.5 Mortality rate due to HCV in thalassemic patients

The most common co-morbidities in thalassemic patients were endocrine diseases, followed by cardiovascular, hepatic and renal diseases.^13^ Since most liver problems are HCV-related, this infection is regarded as the main cause of mortality in 40.5% of patients, as confirmed by some experts.

#### 2.2.6 HCV diagnosis and treatment strategies

The HCV treatment rate has significantly increased due to the emergence of new drugs such as DAAs in recent years. Accordingly, different scenarios were considered in our model. Some predictions were made based on different scenarios regarding the rate of diagnosis and treatment. These scenarios were used for assessing the impact of different strategies on HCV eradication.

Scenario 1 was based on IFN treatment, where the number of HCV-infected thalassemic patients was calculated based on the suggested model. The total number of patients in different years was predicted from 2017 to 2030. Diagnosis and treatment rates were 30% and 50%, respectively. Scenario 2 assumed base case scenarios until 2017, and IFN-based treatments were replaced with DAAs with a stepwise increase in uptake from 2017. The rate of diagnosis was fixed, while the rate of treatment increased stepwise from 50% in 2017 to 95% in 2030. Scenario 3 was similar to Scenario 2 regarding the increase in diagnosis and treatment rates with both diagnosis and treatment rates increasing to 50% and 90% from 2017, respectively. Scenario 4 considered the advent of DAAs, where both diagnosis and treatment rates increased from 2017. According to expert opinion, this scenario assumed an increase in diagnosis and treatment rates up to 90% and 95%, respectively.

## 3 Results

In the present study, all descriptive, cross-sectional and group studies about HCV prevalence in thalassemic patients were considered in order to estimate prevalence. Articles were evaluated in 3 steps by studying their title, abstract, and full-text. Thirty-two out of 67 high-quality articles published from 1996 to 2015 were included in the analysis, while 35 articles were excluded because of insufficient information, incomplete title or abstract or unavailability of the full-text manuscript. In addition, to avoid repetitive data, studies performed in the same province by the same author were excluded.

**Fig. 1:**
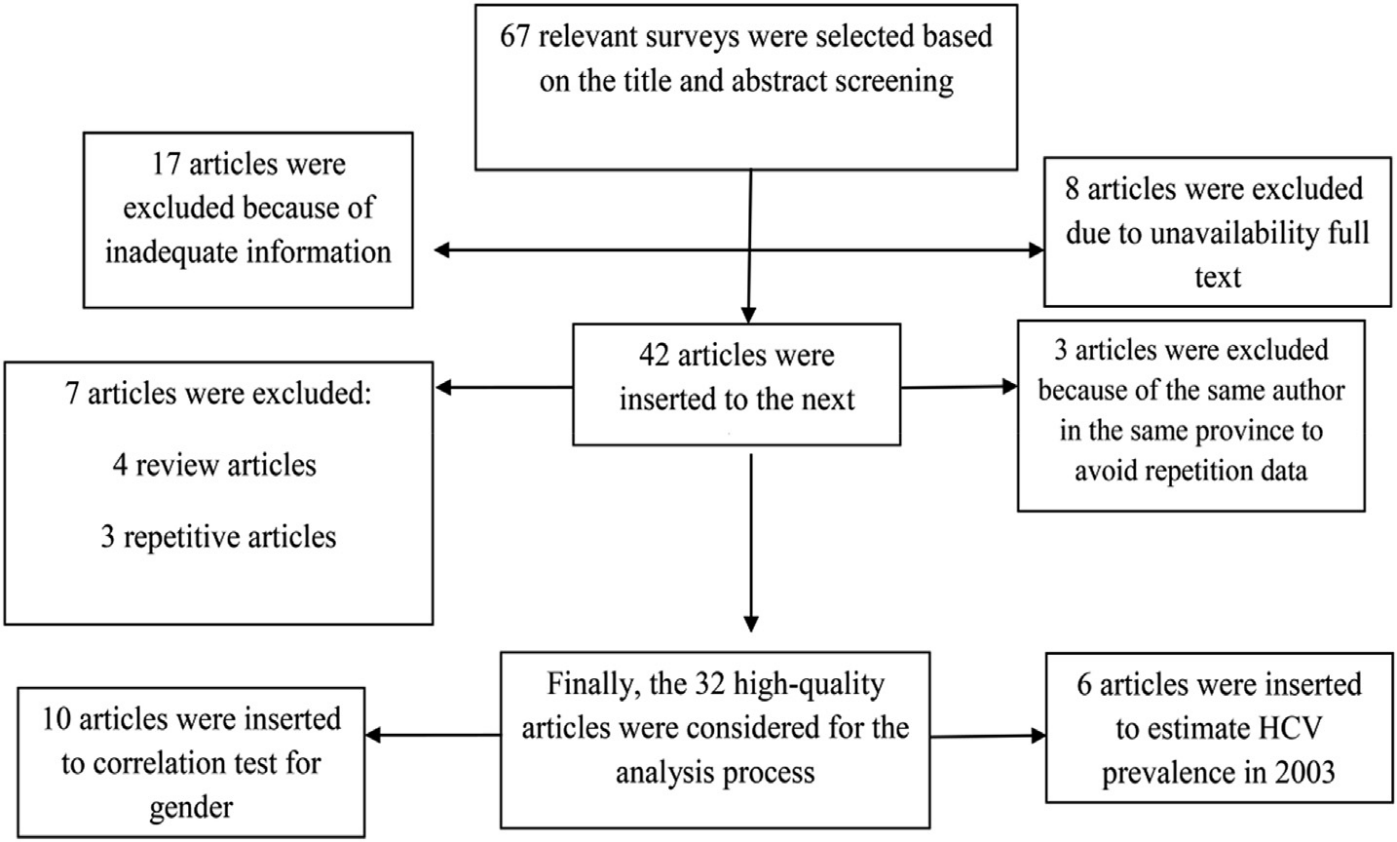
Flowchart of articles selected for systematic review and meta-analysis.

In order to evaluate the impact of gender on the rate of HCV infection in thalassemic patients, 10 articles were examined, as 22 articles reported inadequate information. The HCV prevalence was estimated to predict the number of HCV-infected thalassemic patients. After determining the quality of studies, type of information, including the first author's name, year of publication, study year and location, sample size, number of patients, gender, and prevalence of HCV in thalassemic patients, data was extracted based on a systematic review and meta-analysis. Fig. 1 displays the detailed search process.

In addition, the Chi-square test was performed to determine the effect of gender on the rate of HCV infection in thalassemic patients. According to categorized data presented in these articles, no relationship was observed between gender and HCV infection in 2488 patients, including 416 HCV-infected patients (202 males and 214 females) and 2072 uninfected patients (1044 males and 1028 females) (p = 0.52). Therefore, the null hypothesis was rejected at a significance level of 0.05 and we concluded that it was not necessary to categorize data based on gender in order to estimate parameters.

**Fig. 2:**
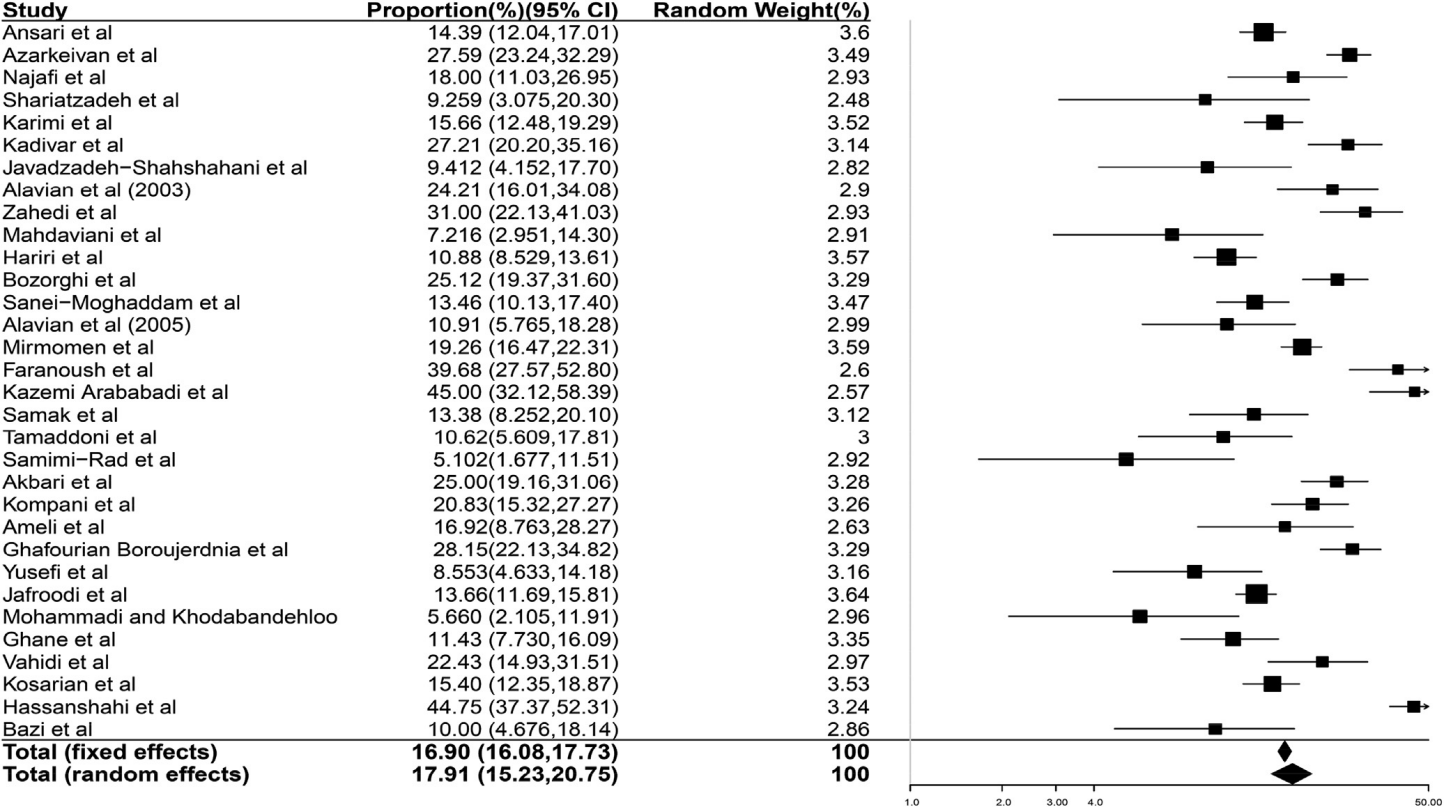
Overall rate of HCV infection in thalassemic patients in Iran (1996–2015). Pooled estimate of positive anti-HCV serostatus in Iran was 16.9% (95% CI 16.08–17.73%) in the fixed effect model and 17.91% (95% CI 15.23–20.75%) in the random effect one based on the inverse variance method.

Since HCV prevalence was considered as the main outcome in this study, binomial distribution was measured to determine variance at a confidence level of 95%. Moreover, the random weighted average was calculated to combine prevalence rates in various studies. Each study was assigned a weight corresponding to its inverse variance. Among 8007 thalassemic patients, 1388 (17.3%) were positive for HCV Abs. The pooled estimate of positive serostatus was 16.9% (95% CI: 16.08–17.73%) in the fixed effect model and 17.91% (95% CI: 15.23–20.75%) in the random effect one according to the inverse variance method. Studies were heterogeneous regarding indices of Q(31) = 303.91 (p < 0.05) and I-squared = 89.80% (95% CI: 86.70–92.18%). Fig. 2 presents point estimates and 95% CI.

**Fig. 3:**
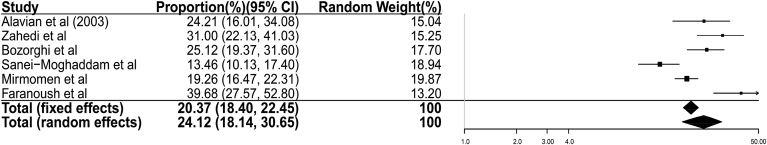
Overall rate of HCV infection in thalassemic patients in Iran (2003–2006). Pooled estimate of positive HCV serostatus in Iran was 20.37% (95% CI 18.40–22.45%) in the fixed effect model and 24.12% (95% CI 18.14–30.65%) in the random effect one with the inverse variance method.

The R program and SPSS version 20 were used for data analysis. Additionally, data were analyzed during 2003–2006. Findings showed that 321 (20.56%) cases were HCV Abs positive from a total of 1561 thalassemic patients. The pooled estimate of positive HCV serostatus in Iran was 20.37% (95% CI: 18.40–22.45%) in the fixed effect model and 24.12% (95% CI: 18.14–30.65%) in the random effect one, based on the inverse variance method. Studies were shown to be heterogeneous regarding indices of Q(5) = 34.44 (p < 0.05) and I-squared = 85.48% (95% CI: 70.36–92.89%). Fig. 3 displays results obtained during this period.

The diagnosis of HCV infection based on antigen-antibody methods, such as ELISA, is widely used in laboratories. Amplification methods such as reverse transcription-polymerase chain reaction (RT-PCR) are recommended; sensitive and specific classic molecular biology techniques are not useful due to the limited number of blood viral RNAs. Therefore, the correction coefficient was used to estimate HCV prevalence more accurately in 2003. It was calculated by comparing ELISA and RT-PCR techniques^45^ as the proportion of positive samples on RT-PCR to ELISA was 82.75% (95% CI: 77.20–88.30%). Consequently, HCV prevalence was changed to 19.96% (95% CI: 15.01–25.36%) in 2003.

In addition, a forward and backward model was used to estimate the number of thalassemic patients. It was based on the number of births and deaths occurring each year. In this model, the number of deaths was set at 1.02 per 100 people and the number of births predicted. Since the number of thalassemic patients born during 1996–2009 and the total number of infants were available for these years,^13^ the thalassemia birth rate could be calculated for this period. In order to determine the birth rate, the total number of births in the country was predicted using the National Organization for Civil Registration dataset. For this purpose, the Holt's model was used in the time series in SPSS to predict the total number of births during 2018–2030.

**Fig. 4:**
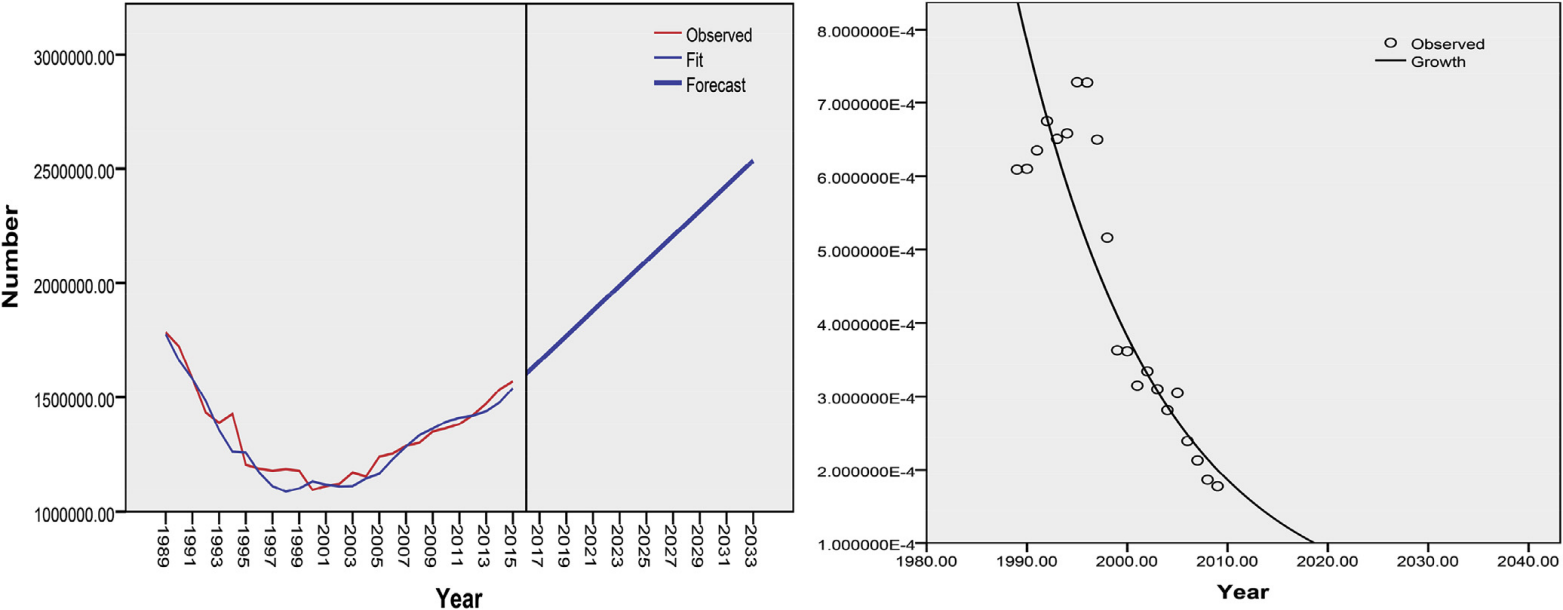
Prediction of total birth in the general population and birth rate in thalassemic patients (Left to right).

The birth rate of thalassemic patients was predicted by the growth model, whereby the coefficient of determination was 0.88. Accordingly, the total number of thalassemic patients was predicted based on the total birth rate as well as the predicted population growth rate. Fig. 4 demonstrates the predictive number of total births in the general population and that of thalassemic patients by 2030. Results indicate that screening plays a significant role in the birth rate of thalassemic patients. Annual birth rate in these patients was predicted to be less than 57 per 10^6^ births by 2020, while the estimated number was 160 per 10^6^ births in 2010.

According to a systematic review and meta-analysis in the country during 2003–2006, HCV prevalence in thalassemic patients was estimated at 24.12% in the random effect model, and its corrected value 19.96%. The number of HCV-infected thalassemic patients was estimated at 3599 people in 2003. According to this rate and a recursive mathematical model, the number of HCV-infected patients was then estimated during 2003–2030. Estimations were based on 4 different treatment scenarios with various treatment and diagnosis rates. In the first scenario, treatment and diagnosis rates were set at 50% and 30%, respectively. Based on these assumptions, the number of patients was 313 in 2018 and 61 in 2030. In the second scenario, we assumed that the treatment success had a stepwise increase to 95% from 2017, and the diagnosis rate was fixed at 30% with the advent of DAAs. In this scenario, the number of patients was predicted to be 310 in 2018 and 25 in 2030.

**Fig. 5:**
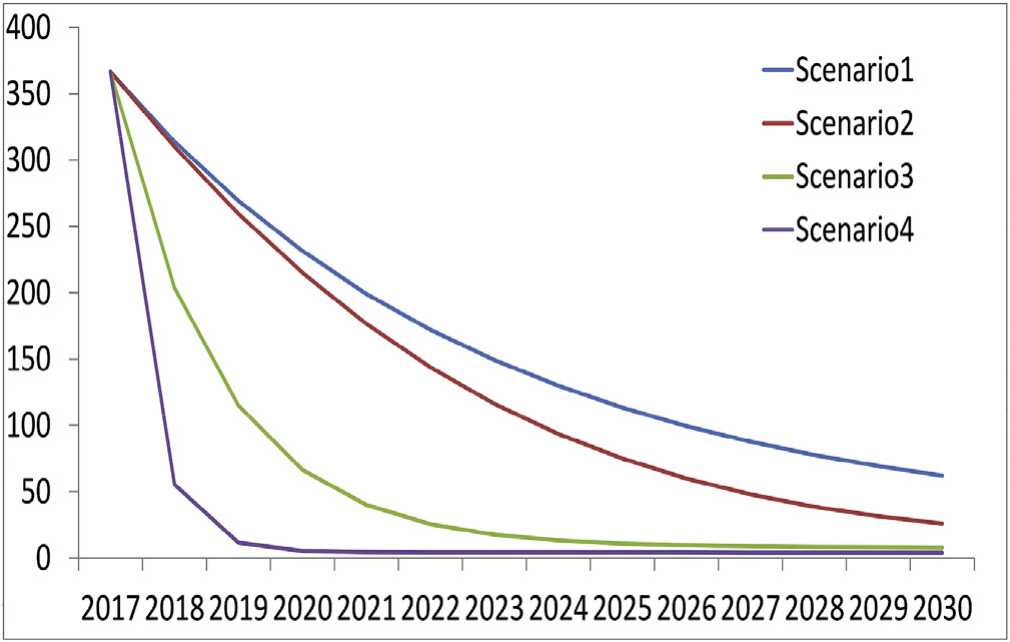
Prediction of the number of HCV-infected thalassemic patients over time in various scenarios.

In the third scenario, the treatment rate was fixed at 90% in 2017 and the assumed diagnosis rate was 50% with the number of patients predicted to be 203 in 2018 and 7 in 2030. The rate of eradication in this scenario was reasonable and HCV was predicted in thalassemic patients, although it was assumed to disappear within a short period. In the final scenario, both treatment and diagnosis rates increased to 90% and 95%, respectively. In this scenario, the number of patients was estimated at 55 in 2018, and it was assumed that HCV infection would be completely eradicated in thalassemic patients by 2020. Table 1 presents a summary of assumptions and estimated input of the model. Fig. 5 demonstrates the prediction curve during this period.

**Table 1:** Model inputs and assumptions.

Input Variable	Value	Year (Reference)
Total thalasssemic population	18500	2007^12^
Thalassemic mortality rate (annual)	1.02 per 100	2015^14^
Corrected HCV prevalence in thalassemic patients	19.96% (95% CI 15.01–25.36%).	2003 [estimated in the current study based on systematic review and meta -analysis]
HCV diagnosis	30%–90%	2018 [expert consensus]
HCV incidence (annual)	2 per 10,000	2018 [estimated in the current study]
Mortality rate caused by HCV in thalassemia	40.5%	2013^5^
HCV treatment rate (annually)	50%–95%	2018 [expert consensus]

Based on our predictions and Scenarios 3 and 4, the number of HCV-infected thalassemic patients will be less than 66 by 2020. With the emergence of DAAs, it is expected to eradicate the infection within a short period. It is worth mentioning that the results are only related to patients who are not resistant to treatment, in other words, the incidence of resistance was not considered in the present study.

## 4 Discussion

A mathematical model was used to predict the number of HCV-infected thalassemic patients over the years. For this purpose, a meta-analysis and systematic review methodology were performed, as the proposed model relied on several parameter estimations. Our findings indicate that the number of patients and eradication time are very different according to four potential treatment strategies.

Due to a lack of comprehensive data on HCV-infected thalassemic patients in Iran, other references were used in the mathematical model. Despite using what we believe to be the most accurate references to estimate parameters, there remain some limitations such as the information related to HCV incidence and diagnostic rates and HCV treatment uptake which was limited. No similar analyses were carried out in this area, therefore, no comprehensive information was available to compare our results.

There are also different therapeutic strategies used in Iran. Some traditional treatments, because of cost or lack of availability of the newer drugs, are still administered to patients although these new types of intervention have an increased rate of success. However, this issue was not considered in our model and treatments for all thalassemic patients were only considered at a fixed rate. Generally speaking, treatment with interferon and ribavirin is performed over a 24-48-week period but have a lower response rate and more side-effects than the new DAAs which have dramatically changed the landscape of HCV treatment for the better. Current guidelines in Iran provide updated recommendations for the clinical management of HCV infection. The first generation of DAAs, such as protease inhibitors, has many side-effects and drug interactions and has been superseded by sofosbuvir with a shorter duration of therapy and better tolerability.^46^

With the introduction of the next DAA generation, sustained virological response has improved significantly with treatment and diagnostic rates as described in Scenario 4, reflective of the therapeutic performance in Iran. It seems that HCV infection could be eradicated in thalassemic patients in the near future in Iran, as it has been in some provinces such as Alborz, Lorestan, and South Khorasan.

## Declaration of competing interest

The authors declare no conflict of interest.
